# Factors Affecting Choice of Childbirth Place among Childbearing Age Women in Western Ethiopia: A Community-Based Cross-Sectional Study

**DOI:** 10.1155/2020/4371513

**Published:** 2020-04-25

**Authors:** Tariku Tesfaye Bekuma, Belaynesh Firrisa, Melese Girmaye Negero, Gemechu Kejela, Haile Bikila

**Affiliations:** ^1^Department of Public Health, Institute of Health Sciences, Wollega University, Ethiopia; ^2^Department of Nursing, Nekemte Specialized Hospital, Ethiopia

## Abstract

**Background:**

Access to proper medical attention and hygienic conditions during delivery can reduce the risk of complications and infections that may lead to serious illness or death or for the mother, baby, or both. In Ethiopia, the high maternal mortality rate with delivery by unskilled birth attendants shows low utilization of maternal health services.

**Objective:**

This study was aimed at assessing factors determining the choice of childbirth place among women of childbearing age in Jimma Arjo District.

**Method:**

A cross-sectional design was conducted in Jimma Arjo District, East Wollega Zone, Ethiopia, from March 20 to April 20, 2018. Multistage sampling technique was used to select a total sample of 506 participants. Data were collected using structured questionnaires to interview women of childbearing age with two trained data collectors. Data was entered into Epi Info and exported to SPSS software version 20 for analysis. Data was checked for its completeness, cleaned, entered, and analyzed accordingly. Bivariate and multivariable data analyses were used to examine factors affecting choice of childbirth place.

**Results:**

A total of 506 women participated in this study, giving a response rate of 97.8%. The study investigated that home delivery was found to be 200 (39.5%)in the study area. Factors found to be statistically associated with choice of institutional delivery at *p* < 0.05 were history of obstetric difficulties (AOR = 6, 95%CI = (2.08, 17.60)), woman educational status (AOR = 4.4, 95%CI = (1.47, 13.42)), husband educational status (AOR = 4, 95%CI = (1.43, 11.60)), two or more ANC visits (AOR = 4, 95%CI = (1.95, 8.52)), and accessibility to vehicle transportation (AOR = 2.8, 95%CI = (1.23, 6.46)).

**Conclusion:**

Preferring health facility as the birthplace in this study seems relatively better compared to other studies. It is shown that both mothers and their husbands attending secondary and greater educational level, history of obstetric difficulties, two or more ANC visits, and physical accessibility to health care facility have influenced mothers to prefer a health institution as the childbirth place. Therefore, any programs aimed at increasing the choice of institutional delivery should work on increasing ANC attendance and transportation facilities in the study area.

## 1. Background

Globally, there was an estimated 289,000 maternal deaths in 2013, yielding a maternal mortality rate (MMR) of 210 maternal deaths per 100,000 live births. Developing countries account for 99% of the global maternal death [1]. Maternal mortality is the highest by far in sub-Saharan Africa, where the lifetime risk of death from pregnancy-related conditions is 1 in 16, compared with 1 in 2800 in rich countries [2, 3]. In Ethiopia, according to EDHS 2011 and 2016, there are 676 and 412 maternal deaths for every 100,000 live birth, respectively [4].

Maternal death is the most extreme consequence of poor maternal health outcomes. However, due to inadequate care during pregnancy and delivery or the first critical hours after birth, more than 30 million women in developing regions suffer from serious diseases and disabilities. These diseases and disabilities include uterine prolapse, pelvic inflammatory disease, fistula, incontinence, infertility, and pain during sexual intercourse. The majority of these deaths and complications could be avoided by access to basic maternity care and improved delivery care, which is supported by adequate medical and surgical cares [5].

Access to proper medical attention and hygienic conditions during delivery can reduce the risk of complications and infections that may lead to death or serious illness for the mother, baby, or both. In Ethiopia, the high maternal mortality rate with delivery by unskilled birth attendants shows low utilization of maternal health services which could be explained by low institutional delivery ranging from as low as 3.7% in Amhara to as high as 79% in the capital city, Addis, showing greater disparity existing within the country [6]. The percentage of live births delivered by a skilled provider remained virtually unchanged for a period of 5 years after 2000, but increased substantially after 2005, from 6% in the 2000 and 2005 EDHS to 10% in 2011 EDHS, and reaching 28% in 2016 EDHS [3, 4, 7].

**Figure 1:**
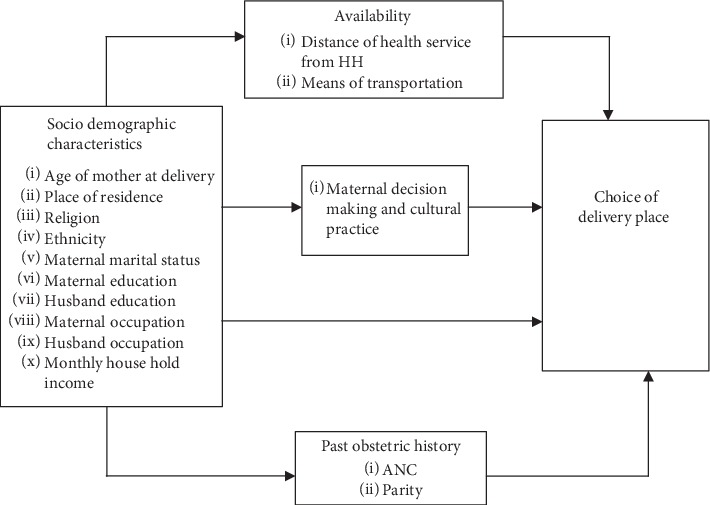
Conceptual framework for analyzing the determinants of maternal choice of birthplace (adapted from Sayih [19]).

As many studies conducted in the country indicated, factors include maternal age, parity, education and marital status, household factors including family size, household wealth, and community factors including socioeconomic status, community health infrastructure, residence, available health facilities, distance to health facilities, poor referral system, and shortage of skilled attendants affect place of delivery, and these factors interact in diverse ways in each context [8–14] (Figure 1).

To improve maternal and child health, many intervention strategies were developed by WHO and other organizations. Similarly, there are different interventions in Ethiopia like focused antenatal care, skilled attendants during delivery, family planning, and expansion and upgrade of comprehensive emergency obstetric care. In addition to these, the government of Ethiopia introduced a health extension program to reach the community level and improve maternal health [15, 16].

Despite all the above global and national efforts in reducing maternal morbidity and mortality through the safe motherhood initiative, there is no significant reduction in developing countries including Ethiopia [17, 18]. Moreover, little has been studied about factors affecting the choice of delivery place among women of childbearing age, in Ethiopia in general and in the study area in particular. Therefore, the aim of this study was to elucidate factors that affect women's choice of place of childbirth in Jimma Arjo District, Oromia regional state, Ethiopia.

## 2. Methods

### 2.1. Study Area and Period

The study was conducted on childbearing age mothers who gave birth to at least one child in Jimma Arjo District of East Wollega Zone, Western Ethiopia, from March 20 to April 20, 2018. Jimma Arjo District is located at 379 km west of Addis Ababa, the capital city of Ethiopia. According to the Jimma Arjo District administration office report, the current (2017) population of Jimma Arjo district was estimated to be 114,175. There are 20 rural kebeles and 2 town administration in the district. There are one district governmental hospital, four health centers, and 20 health posts in the district.

### 2.2. Study Design

A community-based cross-sectional study design was used.

### 2.3. Population

All women of childbearing age (15-49 years) who reside in Jimma Arjo District were the source population, while all women of childbearing age who had given at least one childbirth in the randomly selected kebeles of Jimma Arjo District were the study population for the study.

### 2.4. Eligibility Criteria

Women of child bearing age who gave at least one childbirth within the two years preceding the study period were included in the study, while those who were severely ill at the time of data collection and who lived less than six months in the study area at the time of data collection were excluded from the study.

### 2.5. Sample

The sample size was determined using the single population proportion formula with a design effect of 2. The estimated proportion was 18.8% which is a percentage of women in Oromia who gave birth at health facilities in 2016 [4]. Taking the assumptions of 95% confidence level and 5% margin of error in the single population proportion formula with a nonresponse rate of 10%, a total of 517 women were interviewed in this study.

### 2.6. Sampling Techniques

**Figure 2:**
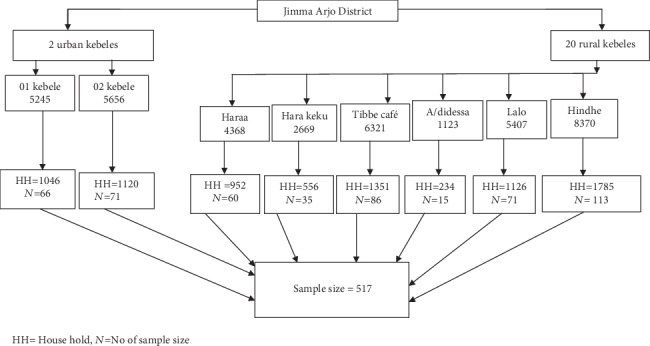
Sampling technique for assessing the determinants of maternal choice of delivery place among childbearing age women of Jimma Arjo District, Oromia, Ethiopia, 2018. HH: household, *N*: no. of sample size.

To get the study respondents, the multistage sampling technique was used. There are a total of twenty rural and two urban kebeles in the district. The two urban kebeles were taken, and from the twenty rural kebeles, six kebeles were selected using the simple random sampling method. The calculated total sample size was distributed across the selected kebeles proportionally to the size of the households within the respective kebeles. Then, the systematic random sampling technique was used to select the actual study participants. First, initial household with eligible woman/respondent was selected randomly, and then, the subsequent households with eligible respondents were selected systematically (Figure 2).

### 2.7. Data Collection and Procedure

A structured questionnaire was prepared in English from different reviewed literatures (29, 32, 36). The questionnaire was translated into Afan Oromo and retranslated to English to ensure consistency. A pretest of the questionnaire was conducted on 5% of the total sample size outside of the study. The data was collected by six trained data collectors and supervised by two suppervisors.

### 2.8. Variables

#### 2.8.1. Dependent Variable

The dependent variable was the woman's choice of birthplace.

#### 2.8.2. Independet Variables

Independent variables include sociodemographic characteristics (age, maternal education, husband education, mother's occupation and husband's occupation, ethnicity and religions, and income), distance from the facility, past obstetric history of the mother, parity, ANC follow-up, obstetric difficulties, maternal decision to choose place of delivery, and means of transportation.

### 2.9. Operational Definition

#### 2.9.1. Choice of Birthplace

The extent at which mothers who gave at least one childbirth preceding the study period preferred the delivery place as either institutional or home due to factors contributing it.

*(1) Traditional Medication*. It is a treatment given culturally to delivery mothers at home to hasten child birth, to relief pain, or to prevent complication of child birth or for others.

*(2) Antenatal Care*. Special care is given to the pregnant mother prenatally (before birth) for prevention or early diagnosis of complications related to pregnancy, delivery, and postpartum.

*(3) Distance*. It is measured in kilometers from home to the nearest health facility. According to the national standard, distance > 5 km from home to health facilities are said to be far. It is measured with a statement, “What is the estimated distance from home to the nearby delivery institution?,” with the options of below 2 km, 2-5 km, and > 5 km.

*(4) Women's Decision on Birthplace*. Women's autonomy or power of women on ones own choice of delivery place is measured with a statement “Who decides on place of your delivery?”

### 2.10. Data Processing and Analysis

After data collection was completed, it was entered into Epi Info version 3.5.4 and exported to SPSS software version 20. It was checked for its completeness, cleaned, and analyzed accordingly. Frequencies and percentages were used to describe the variables. The independent variables which was at *p* value < 0.25 with the dependent variable in a bivariate analysis were included in the multivariate logistic regression model. In the multivariate analysis, the association of the independent variable with the outcome variable was considered to be statistically significant at *p* value of < 0.05 with the corresponding 95% confidence interval.

### 2.11. Data Quality Management

To manage the quality of data, a two-day training was given to the six data collectors and a one-day training to two supervisors on the study tools and how to collect the data. Pretest of the questionnaires was done on 5% of the sample size, and the appropriate correction was made before data collection. All collected data were checked for completeness, accuracy, and consistency by the supervisor every day, and onsite close supervision and technical support was given by supervisors and principal investigator.

### 2.12. Ethics and Consent to Participate

Ethical clearance was obtained from the research and ethics review committee of Wollega University, and official permission was granted from the Jimma Arjo District health office before data collection. Objective of the study was explained to in detail. The respondents were reassured about the confidentiality of their response; their voluntarily participation was ensured; and right to take part or terminate at any time they wanted was respected. Written informed consent was taken from all of them before involvement in the study.

## 3. Results

### 3.1. Sociodemographic Characteristics of the Respondents

A total of 506 women participated in this study giving a response rate of 97.8%. Out of 506 respondents, 116 (22.9%) were from urban while 390 (77.1%) were from rural kebeles. The age of mothers ranged from 20 to 45 years with mean and standard deviation (SD) of 31.6 ± 6.4 years. Majority of the mothers were between 25 and 29 years, while those above the age of 40 years were the least, 58 (11.5%). Three hundred ninety-five (78.9%) of respondents from the study area were married, while 92 (18.2%) were divorced. 453 (89.5%) of the respondents were Oromos in ethnicity. Regarding religion, majority of participants (308 (60.9%)) were protestants followed by Orthodox 182 (36%).

Concerning occupational status of the respondents, majority (302(59.7%)) of them were housewives, followed by merchants 75 (14.8%), while the least (6(1.2%)) were students. In their educational status, 168 (33.2%) of respondents were able to read and write, 151(29.8%) cannot read and write, whereas 61 (12.1%) of them were diploma and above(Table 1).

### 3.2. Past Obstetric Characteristics of the Repondents

Three hundred six (60.5%) of the study subjects were between parity 2 and 4, followed by a parity greater than four 88 (17.4%), while 112 (22.1%) were parity one. Three hundred sixty-two (71.5%) of mothers had ANC follow-up at a health institution, while 144 (28.5%) of mothers had no. The result also showed that 85 (16.8%) of mothers were prone to obstetric difficulties (Table 2).

### 3.3. Women's Choice of Delivery Place

**Figure 3:**
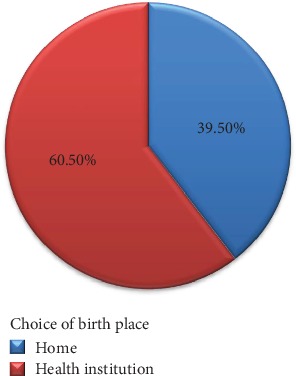
Maternal choice of delivery place in Jimma Arjo District, Oromia, Ethiopia, 2018.

Of the total respondents, 200 (39.5%) chose home whereas the rest of respondents (306(60.5%)) chose health institution as a birthplace (Figure 3).

**Figure 4:**
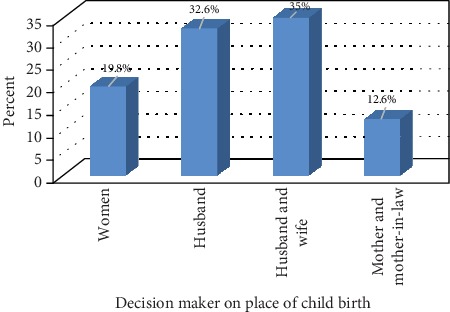
Woman decision making powering Jimma Arjo District, Oromia, Ethiopia, 2018.

Pertaining to a decision made on place of childbirth, 177 (35%) of respondents replied that both husband and wife made a joint decision, 165 (32.6%) by their husband, and 100 (19.8%) by women themselves (Figure 4).

In this study, it was reported that nearly half (251 (49.6%)) of the respondents went on foot, followed by on vehicle 164 (32.4%) and traditional transportation by mule/horse/karezza 91 (18%) to reach the nearby facility.

**Figure 5:**
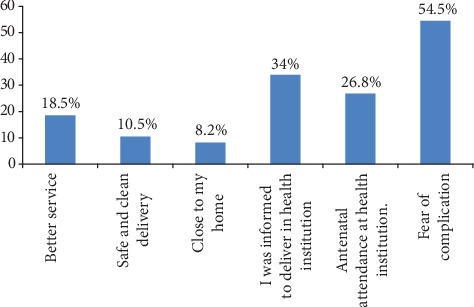
Reasons for preferring institutional delivery (*n* = 306) in Jimma Arjo District, Oromia, Ethiopia, 2018. ^∗^More than one possible answer was used.

Concerning reasons for choosing institutional delivery, 161 (54.5%) of them mentioned due to fear of complications, followed by informed to deliver in a health institution 81 (34%), while the least (8.2%) reported due to closeness of health institution to their home (Figure 5).

**Figure 6:**
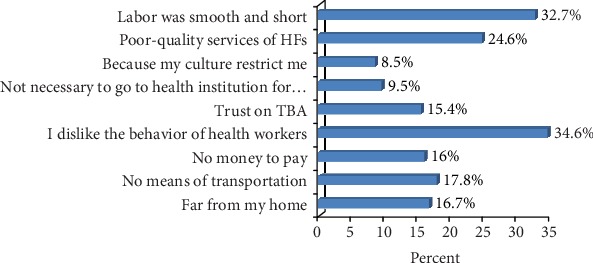
Reasons for preferring home delivery (*n* = 200) in Jimma Arjo District, Oromia, Ethiopia, 2018. ^∗^More than one possible answer was used.

The respondents were also asked to justify why they chose home delivery, and the reasons stated were disliking behavior of health workers 74 (34.6%), lack of money to pay for transportation and health service-related costs 68 (16%), and the least mentioned reason was having trust on TBA 58 (15.4%) (Figure 6).

Regarding assistance of the last childbirth at the health institution, 182 (59.7%) were assisted by midwives, followed by health officers 72 (23.6%) and nurses 36 (11.8%). From those mothers who gave their last birth at home, 95 (47.3%), 73 (36.3%), and 26 (12.9%) of them were assisted by their neighbors, mothers-in-law, and their mothers, respectively (Table 3).

### 3.4. Factors Affecting Choice of Childbirth Place

In bivariate logistic regression analysis, variables with *p* value < 0.25 which were added in the final multivariate logistic regression model were age of respondents, residence, educational status, husband education, monthly household income, information of delivery in a health institution, ANC visits, obstetric difficulties, distance to the nearby health institution, means of transportation, and time spent to reach health institution.

Accordingly, a multivariate logistic regression analysis was done and the result showed that mothers who completed secondary education were 4.4 times more likely to choose health institution as childbirth place compared to those who cannot read and write (AOR = 4.4 (1.47, 13.42) at 95% CI). Similarly, women whose husband completed secondary education were 4 times more likely to choose health institution as delivery place compared to a husband who cannot read and write (AOR = 4 (1.43-11.60) at 95% CI). Compared to mothers who had first ANC visit, those mothers who had 2-3 times ANC follow-up were also 4 times more likely to choose health institution as a delivery place (AOR = 4 (1.95, 8.52) at 95% CI). It was also found that a woman who have ever encountered obstetric difficulties and who had access to vehicles to reach health institutions was more likely to choose institutional delivery with adjusted odds ratio of (6, **(**2.08, 17.60**)** at 95% CI) and (2.8 **(**1.23, 6.46**)** at 95% CI), respectively (Table 4).

## 4. Discussion

This study revealed that 200 (39.5%) of the total respondents chose home delivery, whereas the rest (60.5%) chose institutional delivery. This finding is almost similar with the study done in Jimma Zone, Southwest Ethiopia, in which 35.38% of the mothers chose home delivery [20]. However, it is inconsistent with EDHS 2016 where home delivery accounts 81.2% [4].This could have resulted from the fact that EDHS is a large-scale national data, while this study was conducted only in a single district.

The reasons mentioned for preferring home delivery were smooth and short labor (32.7%), uncomfortable behavior of health workers (34.6%), no means of transportation (17.8%), and no money to pay for transportation and health service (16%). Though the magnitude differs, similar reasons were also mentioned in the study conducted in Dega Damot woreda in which no money to pay for transportation and health service (113 (50.7%)), no means of transportation (134 (60.1%)), and smooth and short labor (106 (47.5%)) [19] were mentioned as reasons. This may indicate that the reasons for preferring home delivery are common everywhere except some differences that exist due to socioeconomic, time, and related differences.

Regarding factors affecting choice of delivery place, in this study, it was found that mothers with the level of secondary education and whose husbands' educational status was diploma and above were more than 4 times more likely to choose institutional delivery compared to those who cannot read and write. This is similar with the study done in Dega Damot district of Amhara and Ahferom district of Tigray, Ethiopia [19, 21]. This could have resulted from the reason that an educated husband is expected to have better knowledge and awareness about the advantages of institutional deliveries and seeking modern health care than those who are not.

It was also found that mothers who had 2-3 ANC visits during the last pregnancy were 4 times more likely to choose health institution compared to women who had a single ANC visit. This finding is consistent with a study finding of Sekela district in which mothers who have ANC visits were 4 times more likely to deliver in health facilities than those who did not have ANC visits [22, 23]. This indicates that the more the number of ANC visits, the more opportunity to reinforce health messages which could result in better understanding and compliance by the women.

Concerning obstetric difficulties, those mothers who faced difficulties were 6 times more likely to choose health institution compared to women who have not. This is inconsistent with the study done in Dega Damot woreda, West Gojjam Zone, which could be due to variation in sociodemographic characteristics [19]. Moreover, participants who had access to vehicles to reach health institutions were 2.8 times more likely to choose childbirth at a health institution than women who went on foot (AOR = 2.8, 95%CI = (1.23, 6.46)) which is consistent with other studies [19, 24]. This reveals that a poor transportation system pushes mothers towards home deliveries.

## 5. Limitation of the Study

A limitation of this study is the possibility of *recall bias* related to maternal age, a person assisting at health institution, and obstetrical difficulties.

## 6. Conclusion

Preference of institutional delivery in this study seems relatively better compared to other studies. However, a significant proportion of mothers are still giving birth to their babies at home. This study has shown that attending secondary and more educational level in both mothers and their husbands, history of obstetric difficulties, history of ANC attendance, having 2-3 ANC visits, and access to vehicle for transportation have influenced mothers to prefer a health institution as a childbirth place. Therefore, efforts should be made to improve a girl's education beyond the primary school level which will have a positive impact on the preference of institutional delivery in the long run. All pregnant mothers should also be encouraged to have antenatal care attendance in nearby public health facilities using different means of information dissemination.

## Figures and Tables

**Table 1:** Sociodemographic characteristics of respondents in Jimma Arjo District, Oromia, Ethiopia, 2018.

Variables	Frequency (*n* = 506)	Percent
Age		
20-24	172	34
25-29	123	24.3
30-34	88	17.4
35-39	65	12.8
≥40	58	11.5
Marital status		
Married	399	78.8
Single	8	1.6
Divorced	92	18.2
Widowed	7	1.4
Residence		
Rural	390	77.1
Urban	116	22.9
Religion		
Protestant	308	60.8
Orthodox	182	36
Muslim	16	3.2
Ethnicity		
Oromo	453	89.5
Amhara	44	8.7
Gurage	5	1.0
Tigre	4	0.8
Occupation		
Housewife	302	59.7
Governmental worker	58	11.5
Merchant	75	14.8
Farmer	50	9.9
Daily labor	15	3.0
Student	6	1.2
Mother education		
Cannot read and write	151	29.8
Read and write	168	33.2
Primarily education	36	7.1
Secondary education	90	17.8
Diploma and above	61	12.1

**Table 2:** Past obstetric characteristics of the respondents in Jimma Arjo District, Oromia, Ethiopia, 2018.

Variables	Frequency (*n* = 506)	Percent
Gravidity		
1	92	18.1
2-4	316	62.5
>4	98	19.4
Parity		
1	112	22.1
2-4	306	60.5
>4	88	17.4
ANC		
Yes	362	71.5
No	144	28.5
Total	506	100
No. of visit of ANC (*n* = 362)		
1	138	27.3
2-3	184	36.4
≥4	40	7.9
Total	362	71.5
Ever obstetric difficulties (*n* = 506)		
Yes	85	16.8
No	421	83.2
Measure taken for obstetric difficulties (*n* = 85)		
Nothing	24	28.2
Visited health institution	53	62.4
Massage, herbs, taking soft drinks	8	9.4

**Table 3:** Last delivery assistance among the woman respondents, in Jimma Arjo District, Oromia, Ethiopia, 2018.

Variables	Frequency (n = 506)	Percent
Assistant at home delivery		
Mother	26	12.9
Mother-in-law	73	36.3
Women from my neighbor	95	47.3
TBA	7	3.5
Assistant at HI delivery		
Midwife	182	59.7
Nurse	36	11.8
Health officer	72	23.6
Doctor	15	4.9
Next delivery place		
Health institution	326	64.4
Home	180	35.6
Husband's choice of place of delivery		
Health institution	298	58.9
Home	208	41.1

**Table 4:** Multivariate analysis of selected variables (*n* = 506) with women's choice of delivery place in Jimma Arjo District, East Wollega Zone, Oromia Regional State, Ethiopia, May 2018.

Variables	Choice of birthplace		COR, 95% CI	AOR, 95% CI
	Home, *n* (%)	HI, *n* (%)		
Residence				
Rural	189 (48.5)	201 (51.5)	1	1
Urban	11 (9.5)	105(90.5)	9 (4.68, 17.23)	2 (0.46, 7.99)
Educational status of the mother				
Cannot read and write	75 (49.7)	76 (50.3)	1	1
Read and write	87 (50.9)	84 (49.1)	0.9 (0.58, 1.40)	0.8 (0.36, 1.86)
Primary education (1–8)	7 (20.6)	27 (79.4)	3 (1.31, 6.72)	1.8 (0.57, 5.93)
Secondary education	22 (24.7)	67 (75.3)	3 (1.71, 5.43)	4.4 (1.47, 13.42)
Diploma and above	9 (14.8)	52 (85.2)	9 (3.67-22.27)	3.8 (0.92, 16.05)
Husband education				
Cannot read and write	60 (58.8)	42 (41.2)	1	1
Read and write	76 (53.1)	67 (46.9)	1.6 (0.91-2.76)	2 (0.77, 5.50)
Primary education (1–8)	27 (42.2)	37 (57.8)	2.5 (1.29-4.91)	1.8 (0.57, 5.60)
Secondary education	25 (24)	79 (76)	3.47 (1.96, 6.15)	4 (1.43, 11.60)
Diploma and above	12 (12.9)	81 (87.1)	12 (5.73, 26.59)	4 (1.17, 13.38)
Information about the benefit of delivery in health institution				
Yes	185 (38.8)	292 (61.2)	1.7 (0.80-3.59)	3.8 (0.98-14.35)
No	15 (51.7)	14 (48.3)	1	
ANC visits (362)				
1	73 (52.9)	65 (47.1)	1	1
2-3	31 (16.8)	153 (83.2)	5.5 (3.33-9.24)	4 (1.95, 8.52)
≥4	16 (40)	24 (60)	1.7 (0.82-3.45)	1.2 (0.45, 3.27)
Obstetric difficulties (like prolonged labor, hemorrhage)				
Yes	6 (7.1)	79 (92.9)	11 (4.80-26.38)	6 (2.08, 17.60)
No	194 (46.1)	227 (53.9)	1	1
Estimated distance to the nearby HI				
<2 km	15 (13.9)	93 (86.1)	4 (2.22-8.09)	0.9 (0.23, 3.94)
2-5 km	90 (51.1)	86 (48.9)	0.8 (0.60-1.29)	1 (0.81, 3.03)
>5 km	95 (42.8)	127 (57.2)	1	1
Means of transport				
On foot	113 (32.9)	230 (67.1)	1	1
Traditional transportation	39 (43.3)	51 (56.7)	1.5 (0.90, 2.34)	1.3 (0.62, 3.09)
Vehicle	48 (65.8)	25 (34.2)	3 (1.92, 5.26)	2.8 (1.23, 6.46)
Time spent to reach HI				
<1 hr	8 (11.8)	60 (88.2)	5.8 (2.73, 12.54)	2 (0.44, 12.49)
1-2 hrs	192(43.8)	246 (56.2)	1	1

## Data Availability

Data will be available on request of the corresponding author.

## Abbreviations

ANC: Antenatal care
AOR: Adjusted odds ratio
CSA: Central Statistical Agency
EDHS: Ethiopian Demographic and Health Survey
MDG: Millennium Development Goals
MMR: Maternal mortality ratio
MCH: Maternal and child health
SBA: Skilled birth attendant
SDG: Sustainable Development Goals
SPSS: Statistical Product and Service Solution
TBA: Traditional birth attendant
UNICEF: United Nations Children's Fund
WHO: World Health Organization.

## References

[1] Adegoke A. A., van den Broek N. (2009). Skilled birth attendance-lessons learnt. *BJOG: An International Journal of Obstetrics & Gynaecology*.

[2] Ohuwole D. (2004). An overview of the maternal and newborn health situation in the African region, in African health monitory. *A magazine of WHO Regional Office for Africa*.

[3] Agency C. S., Macro O. R. C. (2006). *Ethiopia Demographic and Health Survey 2005*.

[4] CSA and ICF International Calverton (2016). *Ethiopia Demographic and Health Survey*.

[5] Gabrysch S., Cousens S., Cox J., Campbell O. (2011). O4-3.1 Distance and quality of care strongly influence choice of delivery place in rural Zambia: a study linking national data in a geographic information system. *Journal of Epidemiology and Community Health*.

[6] World Health Organization (2010). *Maternal mortality*.

[7] Central Statistical Agency Ethiopia (2012). *Demographic Health Survey, 2011*.

[8] Say L., Raine R. (2007). A systematic review of inequalities in the use of maternal health care in developing countries: examining the scale of the problem and the importance of context. *Bulletin of the World Health Organization*.

[9] Gabrysch S., Cousens S., Cox J., Campbell O. (2011). Distance and quality of care strongly influence choice of delivery place in rural Zambia: a study linking national data in a geographic information system. *Journal of Epidemiology and Community Health*.

[10] Samai O., Sengeh P., (The Bo PMM Team) (1997). Facilitating emergency obstetric care through transportation and communication, Bo, Sierra Leone. *International Journal of Gynecology & Obstetrics*.

[11] FMOH, UNICEF, UNFPA, WHO, AMDD (2009). *National Baseline Assessment for Emergency Obstetric and Newborn Care: Ethiopia, 2008*.

[12] Agency C. S., Macro O. R. C. (2006). *Ethiopia Demographic and Health Survey 2005*.

[13] Muleta M., Fantahun M., Tafesse B., Hamlin E. C., Kennedy R. C. (2007). Obstetric fistula in rural Ethiopia. *East African Medical Journal*.

[14] WHO, UNICEF, UNFPA (2014). *The World Bank and United Nation Population Division: Trends in Maternal Mortality: 1990 to 2013*.

[15] Federal Democratic Republic of Ethiopia Ministry of Health (2010). *Health Sector Development Program*.

[16] Prata N., Passano P., Sreenivas A., Gerdts C. E. (2010). Maternal mortality in developing countries: challenges in scaling-up priority interventions. *Women's Health*.

[17] WHO, UNICEF, UNFPA, the World Bank and United Nation Population Division (2014). *Trends in maternal mortality: 1990 to 2013: estimates by WHO, UNICEF, UNFPA, The World Bank and the United Nations Population Division*.

[18] United Nation and the African Union (2013). *Report on Progress in Achieving the Millennium Development Goals in Africa, 2013*.

[19] Sayih A. (2014). *Factors determining the choice of delivery place among women’s of childbearing age in DegaDamot woreda*.

[20] Yegezu R. T., Kitila S. B. (2015). Assessment of factors affecting choice of delivery place among pregnant women in Jimma Zone, South West Ethiopia: Cross Sectional study. *Journal of Womens Health Care*.

[21] Weldearegay H. G. (2015). Factors affecting choice of place for childbirth among women’s in Ahferom woreda, Tigray, 2013. *Journal of Pregnancy and Child Health*.

[22] Shimeka A. (2012). Institutional delivery service utilization and associated factors among mothers who gave birth in the last 12 months in Sekela District, North West of Ethiopia. *BMC Pregnancy and Childbirth*.

[23] Ababulgu F. A., Bekuma T. T. (2016). Delivery site preferences and associated factors among married women of child bearing age in Bench Maji Zone, Ethiopia. *Ethiopian Journal of Health Sciences*.

[24] Lwelamira J., Safari J. (2012). Choice of place for childbirth: prevalence and determinants of health facility delivery among women in Bahi District, Central Tanzania. *Asian Journal of Medical Sciences*.

